# Association of Insulin Resistance with Dysglycemia in Elder Koreans: Age- and Sex-Specific Cutoff Values

**DOI:** 10.3390/jpm15090438

**Published:** 2025-09-15

**Authors:** Sang Min Yoon, Boyoung Park

**Affiliations:** 1 Graduate School of Public Health, Hanyang University, Seoul 04763, Republic of Korea; sm1004041@naver.com; 2 Department of Preventive Medicine, Hanyang University College of Medicine, Seoul 04763, Republic of Korea; 3 Hanyang Institute of Bioscience and Biotechnology, Hanyang University, Seoul 04763, Republic of Korea

**Keywords:** Homeostatic Model Assessment of Insulin Resistance, Homeostatic Model Assessment of Beta-Cell Function, cutoff, elder people, type 2 diabetes mellitus, pre-diabetes mellitus, dysglycemia

## Abstract

**Background/Objectives**: Dysglycemia including pre-diabetes mellitus (Pre-DM) and type 2 diabetes mellitus (T2DM) is associated with insulin resistance. This study aimed to support personalized early diagnosis of dysglycemia by proposing optimal, sex- and age-specific cutoff values for Homeostatic Model Assessment of Insulin Resistance (HOMA-IR) and Homeostatic Model Assessment of Beta-Cell Function (HOMA-β) in Koreans aged ≥65 years. **Methods**: This study analyzed 3862 older Koreans from the 8th Korea National Health and Nutrition Examination Survey data (2019–2021), excluding those with prior diabetes or medication. The participants were classified into normal and dysglycemia groups, based on fasting plasma glucose (FPG) and glycated hemoglobin (HbA1c). Sex- and age-specific optimal cutoff values were determined using Youden’s Index (YI) and area under the curve (AUC). **Results**: For T2DM, the optimal HOMA-IR cutoff was 2.25 for men and 2.03 for women, with strong discriminative performance (AUCs: 0.828 and 0.823, respectively). Stratifying cutoff values further by sex and age improved the diagnostic accuracy (AUC > 0.83 in most subgroups), underscoring the value of tailored thresholds. For pre-DM, the HOMA-IR cutoff was 1.73 in men and 1.85 in women (AUCs: 0.682 and 0.665, respectively). Age- and sex-specific cutoffs modestly improved AUCs, particularly in men (up to 0.7), although the improvement was less consistent among women. HOMA-β showed no significant association with dysglycemia, and no meaningful cutoff values were identified. **Conclusions**: HOMA-IR is a promising marker for the early identification of dysglycemia in older adults when interpreted through a personalized lens. Applying sex- and age-specific cutoff values enhances diagnostic precision and supports a more individualized approach to metabolic risk assessment. Further longitudinal studies are warranted to validate these personalized thresholds and to optimize early detection strategies in diverse populations.

## 1. Introduction

Dysglycemia refers to a spectrum of abnormal blood glucose conditions, including pre-diabetes mellitus (Pre-DM) and type 2 diabetes mellitus (T2DM) [1]. Without timely intervention, these conditions can progress to serious complications, including both microvascular and macrovascular diseases [2,3]. Pre-DM represents a particularly critical window for preventive care, as it often precedes T2DM and independently contributes to adverse outcomes. T2DM, a chronic metabolic disorder accounting for 90–95% of all diabetes cases, is characterized by insulin resistance and relative defects in insulin secretion. It is frequently asymptomatic in its early stages, leading to delayed diagnosis and missed opportunities for early intervention [2].

Insulin resistance plays a central role in the development of a wide range of cardiometabolic disorders, including atherosclerosis, and is considered a key mechanism underlying dysglycemia [4]. Among the various methods used to assess insulin resistance, the Homeostatic Model Assessment of Insulin Resistance (HOMA-IR) is a widely accepted and accessible surrogate, calculated using fasting plasma glucose (FPG) and insulin levels [4]. Although the gold standard for measuring insulin resistance is the hyperinsulinemic-euglycemic clamp (HEC) test, HOMA-IR shows strong correlation with HEC while being more feasible for use in population-level studies [5]. However, emerging evidence suggests that HOMA-IR values vary significantly with individual characteristics, particularly age and sex. For example, HOMA-IR increases substantially after age 50 in non-diabetic women, and sex-based hormonal differences may influence insulin sensitivity [6]. Similarly, the Homeostatic Model Assessment of Beta-Cell Function (HOMA-β)**,** which estimates pancreatic beta-cell function based on the same fasting indices, also demonstrates variability across subgroups, particularly with age [7].

Importantly, prior research has highlighted the limitations of applying uniform HOMA-IR cutoffs across age groups, especially in older adults. A study in the Korean population showed that HOMA-IR had reduced diagnostic accuracy for dysglycemia in individuals aged ≥65 years—particularly among women—when using cutoff values developed for younger populations [8]. Additionally, the discriminative power of HOMA-β to differentiate between diabetic and non-diabetic individuals decreases with age [9]. These findings underscore the need for a personalized approach to screening and risk stratification—one that accounts for age- and sex-specific physiological differences. Rather than relying on generalized cutoffs, tailoring diagnostic thresholds for HOMA-IR and HOMA-β could improve early detection of insulin resistance and enable more effective prevention of dysglycemia in high-risk subpopulations. Therefore, this study aims to determine optimal sex and age-specific HOMA-IR and HOMA-β cutoff values in Korean adults aged ≥65 years, to better identify individuals at high risk of developing Pre-DM and T2DM. By adopting a stratified and personalized approach, this study seeks to contribute to more precise and equitable risk prediction in older populations.

## 2. Materials and Methods

### 2.1. Study Design and Population

This study used data from the 8th Korea National Health and Nutrition Examination Survey (KNHANES) conducted from 2019 to 2021. The KNHANES is a population-based cross-sectional survey designed to assess the health behaviors, health status, and nutritional status of the Korean population [10]. Each survey was conducted by specially trained interviewers, and the data were collected through direct and standardized physical examinations in specially equipped mobile examination centers [10]. A representative sample of non-institutionalized civilians was obtained from all geographic regions of the country [10]. The participants were selected using a rolling cluster sampling design, stratified into multiple stages and proportionate by age [10]. The participants provided written informed consent at enrollment, and the data were received in an anonymized form [10]. The 8th KNHANES was conducted according to the principles expressed in the Declaration of Helsinki [10], and this study was granted an exemption from requiring ethics approval by the Institutional Review Board of Hanyang University (approval number: HYUIRB-202409-016).

**Figure 1:**
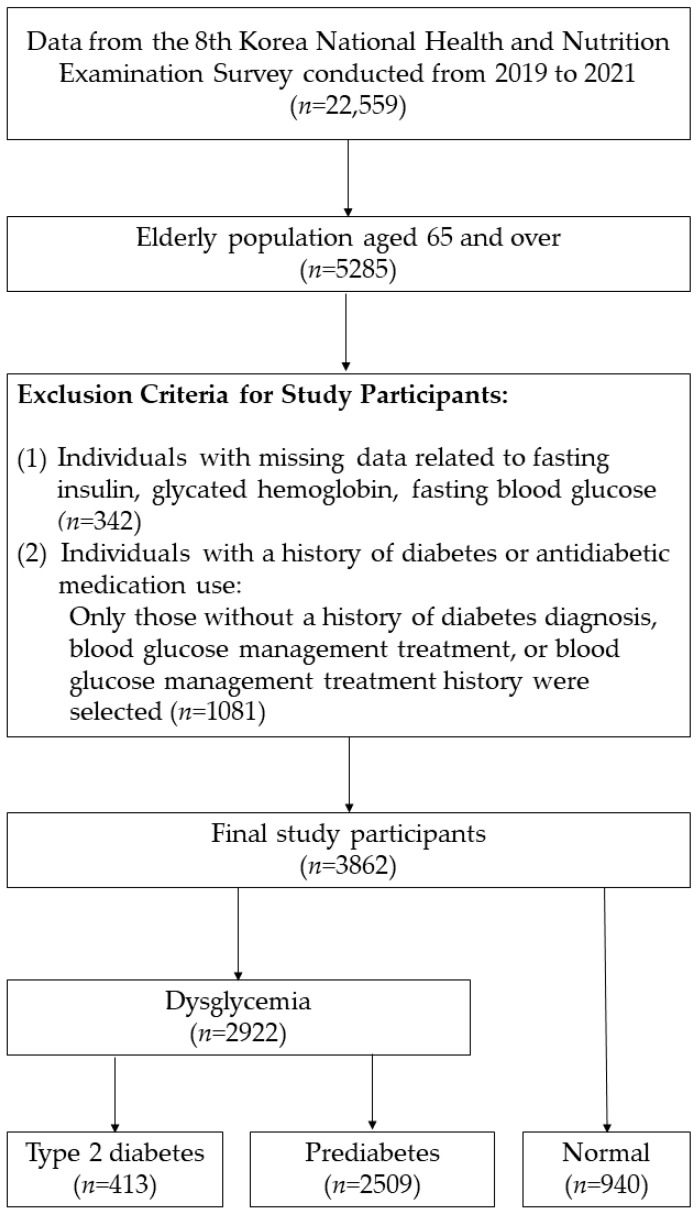
Participant Selection Process.

The analysis was restricted to 5285 individuals excluding 17,274 participants under the age of 65 years from the initial 22,559 individuals aged one year and above. Of 5285 individuals, 342 with missing data on fasting insulin, glycated hemoglobin (HbA1c), and FPG were excluded. Additionally, 1081 individuals with a history of diabetes or antidiabetic medication use were excluded, resulting in a final study population of 3862 individuals. The distribution of disease categories among the study participants is illustrated in Figure 1, with 413 individuals having T2DM, 2509 having pre-DM, and 940 classified as normal (Figure 1).

According to the diagnostic criteria of the American Diabetes Association and the 2021 Korean Diabetes Clinical Practice Guidelines, the final study participants were categorized into three groups: normal, pre-DM, and T2DM [2,3]. The diagnostic criteria for diabetes were based on FPG and HbA1c levels [2,3]. Normal status was defined as having an FPG < 100 mg/dL and an HbA1c < 5.7%, pre-DM was defined as having an FPG of 100–125 mg/dL or an HbA1c of 5.7–6.4%, and T2DM was defined as an FPG ≥ 126 mg/dL or an HbA1c ≥ 6.5% [2,3]. Dysglycemia in this study encompassed both pre-DM and T2DM.

### 2.2. General Characteristics and Anthropometric Factors

The study examined sociodemographic and lifestyle factors such as sex, age, aerobic and strength exercise, smoking habits, education level, heavy alcoholics, household income, and body mass index (BMI).

Age was grouped into three categories: 65–69, 70–74, and ≥75 years [10]. According to the time spent in aerobic exercise, the participants were classified into two groups: those who engaged in at least 2.5 h of moderate-intensity physical activity, 1.25 h of vigorous-intensity physical activity per week, or a combination of both that met the equivalent time requirements, and those who did not meet these criteria [10,11]. Strength exercise was defined as the number of days in the past week on which the participant engaged in strength training exercises such as push-ups, sit-ups, dumbbell/barbell lifting, or pull-ups [10,11]. The number of days of strength exercise was classified into none, 1–3, and ≥4 days. Smoking habits were classified into three categories: non-, former, and current smoker [10,12]. Former smokers were defined as those who had a lifetime smoking history of “5 packs (100 cigarettes) or more” but reported not smoking currently. Current smokers were defined as those with a similar lifetime smoking history who responded that they were “smoking daily” or “smoking occasionally” at the time of the survey [10,12]. Educational levels were classified into four categories: elementary school or below, middle/high school graduation, 2–4-year college graduation, and graduate school or higher [10]. The degree of consumption of alcoholic drinks was classified into three categories according to number of days per week: none, less than once a week, and nearly daily. Heavy alcoholic drinking was defined as consuming ≥7 glasses of drinks (or 5 cans of beer) for men, and ≥5 glasses (or 3 cans of beer) for women [10,13]. Following the 8th KNHANES guideline, all alcohol intake was standardized to the number of “glasses” regardless of beverage type: one can of beer (355 mL) was counted as 1.6 glasses; one bowl of makgeolli (250 mL) was considered equivalent to one glass; and one bottle of soju was counted as seven glasses [10,13]. These rules were applied to compute total alcohol consumption and to determine heavy drinking as defined above [10,13]. Household income was classified into four equal categories based on the equivalized monthly income, calculated by dividing the monthly household income by the square root of the number of household members, and then stratified by sex and age [10]. BMI was classified according to the 2020 guidelines from the Korean Society for the Study of Obesity into three categories: <23, 23–24.9, and ≥25 kg/m^2^ [14]. All blood tests, including insulin, FPG, and HbA1c, as well as anthropometric measurements, were conducted using standardized methods according to the official protocol of the KNHANES [15].

### 2.3. HOMA-IR, HOMA-β Definition

The formulas for calculating HOMA-IR and HOMA-β when FPG is measured in mg/dL were as follows [16]:

HOMA-IR = [Fasting insulin (μU/mL) × Fasting plasma glucose (mg/dL)]/405;

HOMA-β = 360 × Fasting insulin (μU/mL)/[Fasting plasma glucose (mg/dL) − 63].

### 2.4. Statistical Analysis

To identify the basic characteristics of the study participants, they were classified into normal, pre-DM, and T2DM groups. For continuous variables, one-way analysis of variance (ANOVA) and Scheffe’s post hoc test were used to compare the means. For continuous variables that did not follow a normal distribution, ANOVA was performed after log transformation and normality was confirmed again. Since all analyses were performed reflecting the complex sample design method, the representative values were expressed as the mean and standard error. Categorical variables were analyzed using the chi-square test. Multivariate logistic regression analysis was performed to determine whether HOMA-IR and HOMA-β were associated with pre-DM and T2DM; in this analysis, lifestyle factors such as regular aerobic exercise, regular strength training, smoking habits, and excessive drinking, sociodemographic characteristics such as age, education level, and household income, and BMI were used as adjustment variables. To determine the optimal cutoff values of HOMA-IR and HOMA-β for distinguishing pre-DM and T2DM from the normal group, the area under the curve (AUC) of the receiver operating characteristic (ROC) curve was calculated, and the point where the Youden index (YI) maximized was used as the standard [17]. This calculation was performed by dividing the sample into sex and 5-year age groups. In addition, the sensitivity, specificity, positive predictive value (PPV), and negative predictive value (NPV) of the cutoff values were analyzed to evaluate the diagnostic value of pre-DM and T2DM. Statistical analyses were performed using the R software (version 4.4.1, 2024-06-14 ucrt) and SAS 9.4 software (SAS Institute Inc., Cary, NC, USA).

## 3. Results

### 3.1. General Characteristics

The baseline characteristics of individuals in each disease group are summarized in Table 1. The proportions of males in the T2DM, pre-DM, and normal groups were 49.45%, 42.80%, and 42.56%, with no statistically significant differences among the three groups. The mean ages of the three groups were 73.03, 72.87, and 72.74 years, with no statistical differences. For FPG, HbA1c and fasting insulin, the T2DM group had the highest mean values of 128.23 mg/dL, 6.76%, and 15.79 µIU/mL, respectively. The mean of HOMA-IR was 5.28 in the T2DM group, which was higher than the values for the pre-DM (mean, 2.37) and normal (mean, 1.59) groups. However, no significant differences in HOMA-β were observed among the three groups.

**Figure 2:**
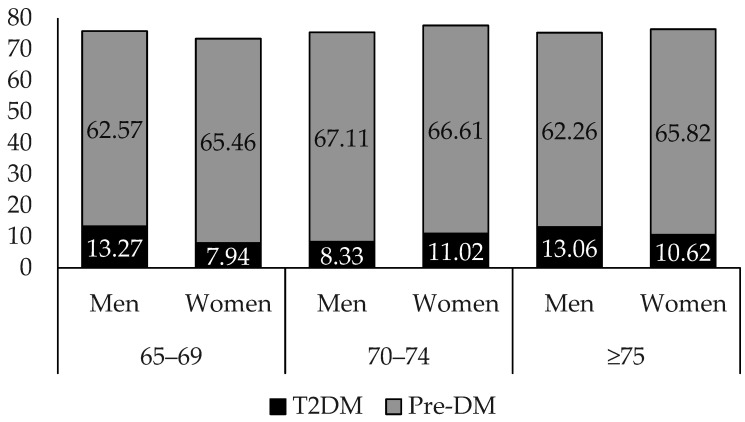
Prevalence of Dysglycemia (T2DM+Pre-DM) according to sex.

Figure 2 presents the prevalence of T2DM and pre-DM across different age groups (65–69, 70–74, and ≥75 years) and by sex. Regarding T2DM, the prevalence rates among men were 13.27% in the 65–69 and 13.06% in the ≥75-year-old group, both higher than those for women, which were 7.94% and 10.62%, respectively. However, in the 70–74-year-old group, the prevalence rate was higher among women (11.02%) than among men (8.33%). Regarding pre-DM, in the 65–69- and ≥75-year-old groups, the prevalence was higher among women (65.46% and 65.82%) than among men (62.57% and 62.26%). Conversely, in the 70–74-year-old group, the prevalence was higher among men (67.11%) than among women (66.61%).

### 3.2. AUC and Optimal Cut-Off Point of Insulin Resistance Index

Table 2 presents the associations between HOMA-IR and HOMA-β, and T2DM and pre-DM by age group. For T2DM compared with normal group, the odds ratio (OR) for HOMA-IR in men and women aged ≥65 years were 1.87 (95% CI: 1.34–2.62) and 2.31 (95% CI: 1.79–2.98), with AUCs of 0.844 and 0.865, respectively. Compared with normal group, for the pre-DM group, the OR for HOMA-IR in men and women aged ≥65 years were 1.34 (95% CI: 1.07–1.68) and 1.78(95% CI: 1.50–2.12), with AUCs of 0.671 and 0.709, respectively. For T2DM, the AUC was greater than 0.84 across all sex and age groups, and while exhibiting some variability in men, it generally showed a decreasing trend with increasing age in women. The AUC value was lower for pre-DM than for T2DM, at 0.67–0.79. However, HOMA-β did not show an association with T2DM or pre-DM, compared with normal status. Therefore, in further analyses for the optimal cut-off, only HOMA-IR was considered, while HOMA-β was presented in a Supplementary Table (Table S1).

Table 3 presents the optimal HOMA-IR cut-off to distinguish T2DM and pre-DM from normal individuals. For T2DM, the cutoff value for men aged ≥65 years was 2.25, with an AUC of 0.828, sensitivity of 72.40%, specificity of 81.82%, PPV of 66.86%, and NPV of 85.40%. For men in specific age groups, the cutoff values were 1.62 for those aged 65–69 years, 2.28 for those aged 70–74 years, and 2.59 for those aged ≥75 years, with an AUC greater than 0.82. For women with T2DM aged ≥65 years, the cutoff value was 2.03, with an AUC of 0.823, sensitivity of 79.36%, specificity of 79.44%, PPV of 59.69%, and NPV of 90.93%. The cutoff values were 1.79 for those aged 65–69 years, 2.08 for those aged 70–74 years, and 1.97 for those aged ≥75 years. The AUC showed an increasing trend with age, with values of 0.819 for the 65–69 age group, 0.842 for the 70–74 age group, and 0.848 for those aged ≥75 years. For pre-DM, the cutoff values for men and women aged ≥65 years were 1.73 and 1.85, with overall AUCs of 0.682 and 0.665, respectively. While the AUCs for pre-DM were generally lower than for T2DM, they showed irregular patterns across all sex and age groups.

## 4. Discussion

In this study, we examined the associations between HOMA-IR, HOMA-β, and dysglycemia—including both T2DM and pre-DM—to identify sex- and age-specific optimal cutoff values for distinguishing abnormal glucose metabolism in Korean adults aged ≥65 years. Among this older population, the optimal HOMA-IR cutoff values for T2DM were 2.06 for men and 2.03 for women, both demonstrating high discriminative performance with AUCs exceeding 0.82. Notably, when cutoff values were further personalized by both sex and 5-year age groups, the diagnostic performance remained consistently high across all subgroups (AUC > 0.81), reinforcing the utility of individualized thresholds. For pre-DM, the optimal HOMA-IR cutoffs were 1.73 in men and 1.85 in women, yielding AUCs below 0.70. While the overall prediction efficacy was more modest than for T2DM, applying tailored cutoffs by sex and age improved performance slightly, with AUCs ranging from 0.66 to 0.75. These findings underscore the value of a precision medicine approach to early detection of dysglycemia, particularly T2DM, by accounting for physiological variations due to sex and aging. In light of previous research reporting decreased accuracy in detecting high-risk T2DM with advancing age—especially among women [8]—our findings further highlight the importance of adopting personalized diagnostic criteria in older adults. By contrast, HOMA-β did not show a significant association with either T2DM or pre-DM, suggesting it may be less useful for risk stratification in this demographic. Overall, these results support the integration of personalized, stratified biomarkers like HOMA-IR into clinical and public health strategies for more effective identification of individuals at high risk for T2DM in aging populations.

A prospective cohort study in China set HOMA-IR cutoff values at 1.4 and 2.0 to distinguish normal glucose tolerance (NGT), dysglycemia, and T2DM in individuals aged 25–74 years [18]. These values were lower than those derived in our study, which were 1.73 for men and 1.85 for women for Pre-DM, and 2.25 for men and 2.03 for women for T2DM. In an Iranian adult population study, HOMA-IR cutoffs for T2DM were 1.85 for women and 2.17 for men aged 20–86 years [19]. Compared with this study, our cutoffs were higher for both sexes, and the sex pattern (men > women) was consistent [20]. In the most relevant study of Korean adults, HOMA-IR cutoffs were 2.87 for men and 2.36 for women for T2DM, and 1.6 for dysglycemia in both sexes [8]. Compared with these findings, our study’s cutoffs were lower for T2DM but higher for Pre-DM [8]. The differences between studies are likely attributable to metabolic changes in older populations, including sarcopenia, body composition alterations, and decreased insulin sensitivity [19,21]. Additionally, reductions in hormones such as IGF-1, testosterone, and myokines exacerbate insulin resistance. These age-related changes may lead to relatively lower cutoff values as insulin resistance increases [21,22], highlighting the need for cutoff values specific to older populations. A previous study in Japan reported that increased HOMA-IR was positively correlated with higher T2DM risk in individuals with impaired insulin secretion [23]. The Japan Diabetes Society recommends a HOMA-IR cutoff of ≥2.5 to define insulin resistance in adults aged 20–79 years [24].

HOMA-IR is a widely used method for assessing insulin resistance, a main risk factor for T2DM and related metabolic disorders. Particularly in the older population, insulin resistance plays a significant role in the development of T2DM. Previous studies have shown that insulin resistance arises when insulin secretion increases but fails to adequately reduce blood glucose levels, thus elevating the risk of T2DM [21,22]. Insulin resistance is also closely linked to cardiovascular diseases making its assessment a critical tool not only for diabetes prevention but also for evaluating overall health [25]. In the older population, insulin resistance is often aggravated by factors such as muscle loss and hormonal changes, which can reduce the predictive power of HOMA-IR [21,26]. Nevertheless, setting the optimal cutoff for HOMA-IR considering age and sex is important because it can reflect individual characteristics of insulin resistance in the older population, and this study also showed good prediction efficacy of HOMA-IR when older-specific cut-offs were applied.

The lack of the association between HOMA-β and T2DM or pre-DM in the older population observed in this study was consistent with the results of another study of the Korean population. Compared with the low HOMA-IR and high HOMA-β groups, the low–HOMA-IR/low–HOMA-β group did not show a significant association with diabetes, but the high HOMA-IR/low HOMA-β group showed the highest risk of diabetes [17], suggesting the high accuracy of HOMA-IR but lower accuracy of HOMA-β for dysglycemia in the Korean population. This finding can be attributed to several factors. While HOMA-β indicates β-cell function, it may fail to adequately evaluate β-cell impairment in older populations where β-cell function declines gradually or has already reached a baseline level. Previous studies have suggested that in East Asian older populations, insulin resistance emerges as the primary contributor to the pathogenesis of T2DM, rather than β-cell function [27]. Second, β-cell function is better assessed through dynamic measures such as glucose-stimulated insulin secretion [7,28]. However, HOMA-β is derived from fasting glucose and insulin levels, which may not fully capture such dynamic responses [7,28]. Third, metabolic changes such as sarcopenia, commonly observed in older adults, may influence both insulin sensitivity and β-cell function [21,26]. However, these factors are not explicitly accounted for in the HOMA-β model [21,26]. Finally, HOMA-β is more indicative of advanced β-cell dysfunction such as near-complete loss of insulin secretion, and may, therefore, have lower sensitivity in detecting early stages of β-cell impairment in diabetes [28,29]. These limitations indicate the potential inadequacy of HOMA-β as a primary marker for assessing T2DM or pre-DM in older Korean populations.

This study has several limitations. First, the definition of dysglycemia and T2DM was based on FPG or HbA1c levels, without considering postprandial glucose levels. Given the relative predominance of postprandial glucose elevation in early T2DM [30], some individuals with impaired fasting glucose may not have been correctly classified as having dysglycemia. However, HbA1c, which was used to diagnose T2DM or prediabetes, is more closely related to post-meal glucose levels than FPG, and we considered HbA1c in combination with FPG when defining T2DM and pre-DM [2,30]. Second, although we excluded participants who were already diagnosed with diabetes or took anti-diabetic medications, the cross-sectional design of the study allowed us to evaluate only the relationship between HOMA-IR and T2DM or pre-DM assessed at the same time point, with limited ability to predict future dysglycemia development. In addition, except diabetes medication, we did not consider other diseases or medications that could affect insulin resistance or insulin function. Third, we adjusted for various sociodemographic and lifestyle factors to estimate the efficacy of HOMA-IR as a marker of insulin resistance and dysglycemia, but there might be other confounding factors that we did not consider.

Despite some limitations, this study obtained significant findings by examining a large sample of older adults residing in Korean communities rather than in hospital settings. It investigated insulin resistance and dysglycemia and introduces sex- and age-specific HOMA-IR cutoff values for a population that has been relatively underrepresented in clinical research. Further longitudinal studies are required to assess the applicability of these cutoff values and to explore their potential in the early identification and prediction of T2DM and dysglycemia across various populations.

## 5. Conclusions

This study aimed to determine sex- and age-specific cutoff values for HOMA-IR in Korean adults aged 65 years and older, with a focus on enhancing personalized risk assessment for dysglycemia. Our findings revealed a strong association between insulin resistance and dysglycemia, and notably, the diagnostic performance of HOMA-IR—measured by AUC—improved when sex- and age-specific cutoff values were applied. This highlights the importance of tailoring diagnostic thresholds to individual characteristics, rather than relying on uniform criteria. These results support the integration of personalized diagnostic approaches into clinical practice for the early detection of pre-diabetes and type 2 diabetes in the older population. By accounting for inter-individual variation in insulin resistance patterns, especially those related to sex and age, these customized cutoffs may help identify high-risk individuals more accurately and enable timely intervention.

Future large-scale, longitudinal studies are essential to validate these personalized HOMA-IR thresholds, assess their predictive value for long-term outcomes, and explore their applicability across diverse populations. Such efforts will further advance precision medicine in metabolic disease prevention and contribute to more effective, equitable healthcare strategies for aging populations.

## 6. Patents

This section is not mandatory but may be added if there are patents resulting from the work reported in this manuscript.

## Figures and Tables

**Table 1:** Baseline Characteristics of T2DM, Pre-DM, Normal.

Characteristics	Dyglycemia (*n* = 2922)				Normal (*n* = 940)		*p*-Value
	T2DM (*n* = 413)		Pre-DM (*n* = 2509)				
	*N*	(W%)	*N*	(W%)	*N*	(W%)	
**Sex**							
Male	195	(49.45)	1051	(42.80)	404	(42.56)	0.086
Female	218	(50.55)	1458	(57.20)	536	(57.44)	
**Age**							
Mean ± SE	73.03	(±0.32)	72.87	(±0.15)	72.74	(±0.22)	0.738
65–69	124	(35.05)	786	(34.08)	313	(36.30)	0.650
70–74	103	(22.36)	699	(25.05)	244	(22.96)	
≥75	186	(42.59)	1024	(40.87)	383	(40.74)	
**Regular aerobic exercise (per week) ^1^**							
Yes	114	(27.57)	701	(28.53)	258	(27.71)	0.213
No	230	(56.46)	1523	(59.80)	567	(60.67)	
Missing	69	(15.96)	285	(11.67)	115	(11.62)	
**Regular strength exercise (per week) ^2^**							
None at all	272	(65.69)	1742	(68.23)	664	(70.29)	0.087
1–3 days	19	(4.45)	173	(7.05)	69	(7.71)	
≥4 days	54	(14.26)	314	(13.37)	98	(11.26)	
Missing	68	(15.60)	280	(11.34)	109	(10.75)	
**Smoking**							
Non-smoker	234	(53.60)	1585	(61.97)	579	(60.86)	0.001
Former smoker	120	(30.92)	655	(26.50)	264	(28.96)	
Current Smoker	44	(12.09)	236	(10.29)	71	(7.27)	
Missing	15	(3.39)	33	(1.25)	26	(2.91)	
**Education**							
≤Elementary	159	(35.30)	1020	(38.59)	375	(36.79)	0.129
Middle–High	144	(39.43)	913	(37.28)	357	(41.46)	
2–4 years college	35	(7.69)	248	(10.59)	78	(8.84)	
≥Postgraduate	7	(1.97)	48	(2.21)	19	(1.97)	
Missing	68	(15.60)	280	(11.33)	111	(10.94)	
**Heavy alcoholics ^3^**							
None at all	301	(71.65)	1974	(78.15)	728	(76.73)	<0.0001
≤1 per week	82	(19.96)	429	(17.53)	175	(19.42)	
Almost every day	17	(5.35)	74	(3.10)	13	(1.18)	
Missing	13	(3.03)	32	(1.22)	24	(2.67)	
**Household income**							
Lower	205	(46.71)	1098	(40.49)	424	(43.96)	0.428
Middle–lower	109	(25.77)	745	(30.13)	266	(27.00)	
Upper middle	60	(15.78)	407	(17.64)	147	(17.16)	
Upper	35	(10.57)	237	(10.69)	96	(11.27)	
Missing	4	(1.16)	22	(1.05)	7	(0.61)	
**BMI (kg/m^2^)**							
Mean ± SE	24.75	(±0.21)	24.02	(±0.07)	23.19	(±0.11)	<0.0001
< 23	119	(30.07)	978	(39.49)	480	(50.24)	<0.0001
23.0–24.9	103	(23.86)	652	(25.72)	232	(25.01)	
≥25	191	(46.07)	879	(34.79)	228	(24.75)	
**FPG, (mg/dL)**							
Mean ± SE	128.23 *†	(±1.35)	100.94 *†	(±0.20)	91.31 *†	(±0.21)	<0.0001
**HbA1c (%)**							
Mean ± SE	6.76 *†	(±0.05)	5.87 *†	(±0.01)	5.43 *†	(±0.01)	<0.0001
**Fasting insulin, (µIU/mL)**							
Mean ± SE	15.79 *†	(±0.90)	9.39 *†	(±0.18)	7.02 *†	(±0.21)	<0.0001
**HOMA-IR**							
Mean ± SE	5.28 *†	(±0.35)	2.37 *†	(±0.05)	1.59 *†	(±0.05)	<0.0001
**HOMA-β**							
Mean ± SE	88.30	(±4.46)	90.65	(±1.96)	89.66	(±2.53)	0.869

The variables are presented as ‘Mean ± Standard Error’ and ‘*N* (Weighted %) T2DM, type 2 diabetes mellitus; Pre-DM, pre-diabetes mellitus; BMI, body mass index; FPG, fasting plasma glucose; HbA1c, glycosylated hemoglobin; HOMA-IR, homeostasis model assessment of insulin resistance; HOMA-β, homeostasis model assessment of beta cell function. * Variables that did not follow a normal distribution were log-transformed to confirm normality before conducting one-way ANOVA. Scheffé’s test was used as the post hoc analysis method. † After log transformation, differences in means were observed among the three groups. ^1^ Regular aerobic exercise was defined as engaging in at least 2 h and 30 min of moderate-intensity physical activity, or 1 h and 15 min of vigorous-intensity physical activity per week, or a combination of both that met the equivalent time requirements. ^2^ Regular strength exercise was defined based on the number of days in the past week participants performed strength training exercises, such as push-ups, sit-ups, dumbbell exercises, barbell exercises, or pull-ups. ^3^ Heavy alcohol consumption was defined as consuming 7 or more glasses (or 5 cans of beer) per occasion for men, and 5 or more glasses (or 3 cans of beer) per occasion for women.

**Table 2:** Odds Ratios and AUC for the Association Between HOMA-IR, HOMA-β and T2DM/Pre-DM from Logistic Regression.

Variable	HOMA-IR			HOMA-β		
	OR (95% CI)	*p*-Value	AUC	OR (95% CI)	*p*-Value	AUC
**Pre-DM**						
**Men**						
Overall	1.34 (1.07–1.68)	0.013	0.671	0.999 (0.996–1.001)	0.321	0.639
65–69	1.34 (0.95–1.88)	0.094	0.704	0.997 (0.992–1.002)	0.195	0.691
70–74	1.40 (0.75–2.62)	0.289	0.742	0.995 (0.991–0.999)	0.017	0.712
≥75	1.38 (0.93–2.06)	0.107	0.719	0.999 (0.998–1.001)	0.375	0.684
**Women**						
Overall	1.78 (1.50–2.12)	<0.0001	0.709	1.001 (0.999–1.002)	0.310	0.606
65–69	2.14 (1.58–2.91)	<0.0001	0.722	0.999 (0.995–1.003)	0.640	0.625
70–74	2.38 (1.58–3.59)	<0.0001	0.794	1.004 (0.999–1.009)	0.090	0.698
≥75	1.60 (1.16–2.20)	0.004	0.719	1.001 (0.999–1.004)	0.265	0.641
**T2DM**						
**Men**						
Overall	1.87 (1.34–2.62)	0.001	0.844	0.995 (0.991–0.999)	0.016	0.717
65–69	1.86 (1.14–3.04)	0.013	0.864	0.985 (0.972–0.998)	0.027	0.852
70–74	3.87 (1.13–13.22)	0.031	0.939	0.976 (0.945–1.009)	0.145	0.828
≥75	2.32 (1.07–5.05)	0.034	0.900	0.999 (0.996–1.002)	0.648	0.754
**Women**						
Overall	2.31 (1.79–2.98)	<0.0001	0.865	0.999 (0.996–1.002)	0.433	0.674
65–69	5.23 (2.89–9.46)	<0.0001	0.939	1.002 (0.996–1.008)	0.552	0.784
70–74	2.81 (1.50–5.25)	0.002	0.915	0.983 (0.966–0.999)	0.041	0.871
≥75	1.58 (1.11–2.26)	0.013	0.853	0.999 (0.996–1.003)	0.724	0.721

HOMA-IR, homeostasis model assessment of insulin resistance; HOMA-β, homeostasis model assessment of beta cell function; OR, odds ratio; CI, confidence interval; AUC, area under the curve; T2DM, type 2 diabetes mellitus; Pre-DM, prediabetes mellitus. All values were adjusted for lifestyle factors (aerobic exercise, strength exercise, smoking habits, and heavy alcoholics) and sociodemographic characteristics (age, education level, household income) and BMI.

**Table 3:** Assessment of T2DM and Pre-DM based on AUC and optimal cut-off points for HOMA-IR.

Variable	Cut-Off	AUC	Sensitivity (95% CI)	Specificity (95% CI)	PPV (95% CI)	NPV (95% CI)
**Pre-DM**						
**Men**						
Overall	1.73	0.682	54.34 (50.96–57.72)	70.23 (64.88–75.58)	83.32 (79.98–86.67)	35.97 (32.01–39.93)
65–69	1.49	0.702	61.65 (55.26–68.04)	60.55 (50.68–70.43)	80.34 (74.52–86.16)	37.65 (29.65–45.66)
70–74	1.69	0.747	62.16 (56.10–68.23)	72.23 (62.67–81.79)	86.67 (81.75–91.60)	39.66 (32.24–47.08)
≥75	1.94	0.700	46.42 (40.72–52.11)	78.19 (71.01–85.38)	85.41 (80.13–90.69)	34.66 (29.49–39.82)
**Women**						
Overall	1.85	0.665	55.77 (52.55–58.99)	73.98 (70.00–77.96)	85.32 (82.97–87.67)	38.15 (34.60–41.71)
65–69	1.82	0.662	53.55 (48.02–59.09)	69.36 (62.09–76.63)	81.43 (76.86–85.99)	37.32 (31.35–43.29)
70–74	1.78	0.700	61.38 (55.85–66.90)	76.27 (68.94–83.60)	88.68 (84.93–92.43)	39.47 (32.44–46.50)
≥75	1.41	0.683	73.09 (69.05–77.12)	60.32 (52.68–67.97)	83.36 (79.75–86.96)	45.19 (38.63–51.75)
**T2DM**						
**Men**						
Overall	2.25	0.828	72.40 (65.24–79.56)	81.82 (77.25–86.39)	66.86 (59.41–74.32)	85.40 (81.26–89.54)
65–69	1.62	0.837	85.86 (76.61–95.10)	63.91 (53.94–73.87)	57.54 (46.45–68.63)	88.80 (81.38–96.23)
70–74	2.28	0.889	65.38 (48.00–82.76)	85.08 (77.60–92.56)	60.83 (43.95–77.72)	87.40 (80.06–94.73)
≥75	2.59	0.865	75.45 (65.97–84.93)	87.43 (81.82–93.04)	76.61 (66.48–86.75)	86.70 (81.04–92.37)
**Women**						
Overall	2.03	0.823	79.36 (73.63–85.09)	79.44 (75.78–83.09)	59.69 (53.46–65.92)	90.93 (88.30–93.57)
65–69	1.79	0.819	79.68 (68.15–91.21)	68.46 (61.18–75.74)	43.11 (31.87–54.34)	91.83 (87.12–96.53)
70–74	2.08	0.842	72.65 (59.84–85.45)	84.58 (78.28–90.89)	69.63 (58.38–80.89)	86.41 (79.51–93.30)
≥75	1.97	0.848	85.72 (78.91–92.52)	79.12 (72.67–85.56)	62.18 (52.09–72.27)	93.26 (89.86–96.66)

HOMA-IR, homeostasis model assessment of insulin resistance; CI, confidence interval; AUC, area under the curve; PPV, positive predictive value; NPV, negative predictive value; T2DM, type 2 diabetes mellitus; Pre-DM, prediabetes mellitus.

## Data Availability

The data analyzed in this study are publicly available from the Korea National Health and Nutrition Examination Survey (KNHANES) and can be downloaded from its official website at https://knhanes.kdca.go.kr/knhanes/main.do (accessed on 19 September 2024).

## Supplementary Materials

The following supporting information can be downloaded at: https://www.mdpi.com/article/10.3390/jpm15090438/s1, Table S1. Assessment of T2DM and Pre-DM based on AUC and optimal cut-off points for HOMA-β.

## Abbreviations

The following abbreviations are used in this manuscript:

Pre-DM	Pre-diabetes mellitus
T2DM	Type 2 diabetes mellitus
HOMA-IR	Homeostatic Model Assessment of Insulin Resistance
FPG	Fasting plasma glucose
HEC	Hyperinsulinemic–euglycemic clamp test
HOMA-β	Homeostasis Model Assessment of Beta-Cell Function
KNHANES	Korea National Health and Nutrition Examination Survey
BMI	Body mass index
ANOVA	One-way analysis of variance
AUC	Area under the curve
ROC	Receiver operating characteristic
YI	Youden index
PPV	Positive predictive value
NPV	Negative predictive value
