# Supplementary material for: Association of Insulin Resistance with Dysglycemia in Elder Koreans: Age- and Sex-Specific Cutoff Values
Source: J Pers Med. 2025 Sep 15;15(9):438. doi: 10.3390/jpm15090438 (PMC12470961; doi:10.3390/jpm15090438)
Supplement: Supplementary file 1 [file jpm-15-00438-s001.zip › jpm-3832608-supplementary.docx]

**Table S1.** Assessment of T2DM and Pre-DM based on AUC and optimal cut-off points for HOMA-β

| **Variable** | **Cut-off** | **AUC** | **Sensitivity  (95% CI)** | **Specificity  (95% CI)** | **PPV  (95% CI)** | **NPV  (95% CI)** |
| --- | --- | --- | --- | --- | --- | --- |
|  |  |  |  |  |  |  |
| **Pre-DM** |  |  |  |  |  |  |
| **Men** |  |  |  |  |  |  |
| **Overall** | 83.25 | 0.625 | 67.17 (63.90-70.45) | 38.66 (33.05-44.28) | 74.99 (71.77-78.21) | 30.08 (25.32-34.84) |
| **65-69** | 60.63 | 0.679 | 27.91 (26.38-29.43) | 61.10 (60.02-62.17) | 79.70 (73.54-85.86) | 34.01 (27.45-40.57) |
| **70-74** | 165.77 | 0.701 | 95.70 (93.42-97.98) | 7.63 (2.18-13.09) | 75.06 (70.36-79.76) | 37.92 (16.07-59.77) |
| **≥75** | 111.72 | 0.666 | 83.48 (79.49-87.46) | 23.62 (15.84-31.40) | 75.04 (70.89-79.19) | 34.19 (23.36-45.03) |
| **Women** |  |  |  |  |  |  |
| **Overall** | 30.32 | 0.580 | 3.32 (2.00-4.65) | 97.85 (96.61-99.09) | 80.73 (69.59-91.87) | 27.18 (24.91-29.45) |
| **65-69** | 135.00 | 0.610 | 86.89 (83.31-90.48) | 19.69 (13.17-26.21) | 73.08 (68.97-77.18) | 37.46 (26.31-48.61) |
| **70-74** | 340.80 | 0.639 | 99.42 (98.73-100.00) | 0.61 (0.00-1.49) | 75.18 (70.70-79.67) | 25.64 (0.00-60.86) |
| **≥75** | 34.97 | 0.608 | 6.74 (4.31-9.16) | 95.44 (92.59-98.30) | 80.09 (68.24-91.93) | 27.35 (23.81-0.88) |
| **T2DM** |  |  |  |  |  |  |
| **Men** |  |  |  |  |  |  |
| **Overall** | 59.25 | 0.711 | 53.49 (45.49-61.49) | 60.32 (55.00-65.64) | 40.58 (33.58-47.59) | 71.91 (66.39-77.42) |
| **65-69** | 60.69 | 0.798 | 57.58 (44.66-70.51) | 66.05 (56.78-75.33) | 49.14 (37.22-61.07) | 73.22 (63.81-82.62) |
| **70-74** | 44.47 | 0.783 | 46.93 (29.61-64.26) | 78.67 (70.34-87.00) | 43.82 (27.40-60.24) | 80.71 (72.16-89.25) |
| **≥75** | 24.71 | 0.703 | 7.84 (0.96-14.71) | 97.19 (94.88-99.50) | 60.34 (29.98-90.71) | 65.89 (59.11-72.67) |
| **Women** |  |  |  |  |  |  |
| **Overall** | 42.55 | 0.703 | 19.92 (14.22-25.61) | 88.12 (84.78-91.46) | 39.15 (28.93-49.37) | 74.14 (70.11-78.17) |
| **65-69** | 33.71 | 0.761 | 22.21 (11.28-33.13) | 95.86 (92.40-99.31) | 61.65 (38.34-84.95) | 80.43 (74.47-86.39) |
| **70-74** | 85 | 0.758 | 60.38 (45.74-75.01) | 39.25 (30.15-48.36) | 32.59 (23.03-42.16) | 67.06 (54.60-79.53) |
| **≥75** | 73.85 | 0.685 | 51.50 (40.36-62.64) | 46.40 (38.25-54.55) | 27.79 (19.96-35.61) | 70.49 (62.22-78.76) |

HOMA-β, homeostasis model assessment of beta cell function; AUC, area under the curve; PPV, positive predictive value; NPV, negative predictive value; T2DM, type 2 diabetes mellitus; Pre-DM, prediabetes mellitus.
